# Evaluation of Nano-Niclosamide in Killing *Demodex folliculorum* In Vitro and the Potential Application in Ocular Surface

**DOI:** 10.3390/pharmaceutics17030332

**Published:** 2025-03-04

**Authors:** Jiani Li, Panqin Ma, Shujia Guo, Danyi Qin, Yuqian Wang, Yuwen Liu, Zixuan Yang, Caihong Huang, Yi Han, Zuguo Liu

**Affiliations:** 1 Xiamen University Affiliated Xiamen Eye Center, Fujian Provincial Key Laboratory of Ophthalmology and Visual Science, Fujian Engineering and Research Center of Eye Regenerative Medicine, Eye Institute of Xiamen University, School of Medicine, Xiamen University, Xiamen 361005, China; lijiani_study@163.com (J.L.); shujiaguoxmu@163.com (S.G.); qindanyidaisy@163.com (D.Q.); 18192175826@163.com (Y.W.); yuwenliu_la@163.com (Y.L.); opthyangzixuan@stu.xmu.edu.cn (Z.Y.); huangcaihong0327@126.com (C.H.); 2 Department of Ophthalmology, The First Affiliated Hospital of University of South China, Hengyang Medical School, University of South China, Hengyang 421001, China; mpqmm46@163.com; 3 Department of Ophthalmology, Xiang’an Hospital of Xiamen University, Xiamen 361005, China

**Keywords:** *Demodex folliculorum*, niclosamide, ocular surface, nanocrystals

## Abstract

**Background/Objectives:** Blepharitis is a condition often caused by *Demodex folliculorum* infestations, resulting in significant ocular discomfort and surface damage. Current treatments offer only temporary relief and fail to eliminate mites effectively. This study evaluates nano-niclosamide (nano-NCL), a lipophilic nanosuspension designed to enhance solubility and permeability, for targeting *Demodex folliculorum*. **Methods:** Nano-NCL was characterized by particle size, zeta potential, transmission electron microscopy, pH measurement, bacterial culture, and HPLC. Viable Demodex mites were collected from patients’ eyelashes and assigned to six treatment groups: DDW, F127, 0.15% nano-NCL, 0.3% nano-NCL, 20% TTO, and Okra. Mite survival was analyzed using Kaplan–Meier curves. The ocular surface safety was assessed via slit-lamp examination, corneal fluorescein staining, and in vivo confocal microscopy. **Results:** The nano-NCL particles are uniformly rod-shaped, approximately 291 nm in size, and exhibit good stability, remaining suspended in various media for up to 20 days. The formulation has a stable pH of 6 and demonstrated no bacterial growth, indicating sterility and suitability for clinical use. In vitro, both 0.15% (*w*/*v*) and 0.30% (*w*/*v*) nano-NCL significantly reduced Demodex survival, with mortality rates ranging from 70.6% to 92.3% within 2 h. Safety evaluations showed minimal corneal staining and inflammation. Notably, 0.15% nano-NCL displayed efficacy comparable to that of 20% tea tree oil (TTO) and Okra, which are established anti-Demodex treatments. **Conclusions:** Nano-NCL, particularly at 0.15%, rapidly eliminates mites while maintaining excellent ocular tolerability, making it a promising treatment for Demodex-related ocular surface diseases.

## 1. Introduction

Blepharitis, a persistent inflammatory disorder impacting the eyelashes, eyelids, and ocular surface, is caused by a variety of etiological factors [1]. Predominantly, it stems from an infestation of Demodex that resides within the eyelid tissue, particularly at the lash bases and within the meibomian glands. *Demodex folliculorum* primarily contributes to blepharitis via direct ocular surface tissue damage, hypersensitivity reactions, and inflammatory responses [2]. The mites feed on ocular surface epithelial cells, consume secretions, and deposit their eggs on the eyelids, leading to the formation of collarettes—a hallmark sign of Demodex-related blepharitis [3,4]. The prevalence of Demodex-related blepharitis is markedly elevated among patients with chronic blepharitis, with a positive correlation observed with advancing age. Research indicates that infestation rates among individuals with blepharitis range from 75.5% to 79.2%, in contrast to rates of 16.2% to 31.4% observed in control groups [5,6,7,8,9].

The current management strategies emphasize the continued use of mechanical debridement of the eyelid margin. Simultaneously, pharmacological treatments, like tea tree oil (TTO), antibiotics, and TP-03 (Tarsus Pharmaceuticals), combined with physiotherapeutic methods, such as hot compresses, massage, and intense pulsed light, have demonstrated a reduction in Demodex mite populations [10,11,12]. Recent studies also highlighted that Okra (*Abelmoschus esculentus* L.), a widely cultivated tropical vegetable, possesses mite-killing effects [13]. However, despite offering varying degrees of temporary symptomatic relief, these interventions have not proven efficacious in completely eliminating the mites or achieving a definitive cure for the disease. Notably, there is a lack of drugs specifically for Demodex-related blepharitis.

Niclosamide (NCL), a derivative of salicylic acid, has been employed for more than four decades in the treatment of tapeworm infections. It works by inhibiting oxidative phosphorylation and augmenting ATPase activity within the cestode mitochondria, ultimately causing the demise of the scolex and proximal segments of parasites [14,15]. Since 1982, this compound has been included on the World Health Organization’s list of essential medicines, reflecting its well-established safety record. Furthermore, accumulating evidence suggests that NCL’s therapeutic potential extends beyond parasitic diseases, encompassing metabolic disorders, immune system diseases, bacterial and viral infections, asthma, arterial constriction, myopia, and cancer [16,17,18,19]. Demodex mites, akin to other eukaryotic organisms, rely on mitochondrial function for their survival. Disruption of this function could lead to energy depletion in the Demodex mites, thereby impairing their fundamental physiological functions, such as movement, reproduction, and nutrient uptake. The need for a highly lipophilic drug to effectively eradicate Demodex mites is primarily due to the lipid-rich environment of the eyelash follicles. The basal part of these follicles, where Demodex mites are located, is surrounded by sebum—a lipid substance that forms a barrier [2]. Lipophilic drugs are more capable of penetrating this sebum layer and diffusing through the lipid-rich follicular lining to reach the mites. This enhanced penetration ensures that the drug achieves an effective concentration at the site of infestation. Therefore, lipophilic drugs are more suitable for treating Demodex-related conditions in the eyelash follicles. However, NCL is a lipophilic drug with a solubility of about 0.1 mg/mL in water at room temperature and a log P of 3.768 at 25 °C, which indicates moderate lipophilicity. Its poor solubility in aqueous solutions limits its effectiveness in ocular applications.

Drug nanocrystals are solid drug particles at the nanoscale, typically ranging from 200 to 800 nm, and are stabilized by a surrounding layer to prevent aggregation [20]. They enhance drug stability and bioavailability, enable targeted delivery to reduce side effects, and offer controlled release for sustained efficacy [21,22]. To surmount this challenge, we have utilized nanocrystal technology to formulate NCL into nano-niclosamide (nano-NCL), thereby dramatically enhancing its solubility and bioavailability. This innovation facilitates a lower dosage and a decreased frequency of administration while amplifying therapeutic effectiveness.

In the current study, nano-NCL was successfully synthesized and thoroughly characterized through assessments of particle size, zeta potential, and electron microscopy imaging. Mite-eradication assays were performed in vitro using two concentrations of nano-NCL (0.15% *w*/*v* and 0.3% *w*/*v*), with the obtained results compared to those of TTO and Okra. Furthermore, a preliminary evaluation of the ocular surface safety of nano-NCL was conducted.

## 2. Materials and Methods

### 2.1. Materials

The materials used in this study include chemicals and reagents such as NCL (Selleck, S3030, Shanghai, China), Pluronic F127 (Aladdin, P477925, Shanghai, China), sodium hydroxide (NaOH, Acmec, S412588, Shanghai, China), phosphate-buffered saline (PBS, Servicebio, G4202, Wuhan, China, pH 7.0–7.4), Dulbecco’s Modified Eagle Medium (DMEM, Gibco, C11995500BT, New York, NY, USA), pH test paper (Beyotime, FPH001, Shanghai, China), and pH calibration buffers (Beyotime, E0763, Shanghai, China). The eye pad soaking solutions from TTO eye pads (20%, YourGa, Shanghai, China) and Okra eye pads (YourGa, Shanghai, China) were utilized in the study. Escherichia coli (Sangon Biotech, NO. B528413, Shanghai, China) was used as a biological material, while fluorescein sodium strips (Tianjin Jingming New Technology Development Co., Ltd., Tianjin, China) and a Carbomer Eye Gel (Bausch & Lomb, Jinan, China) were employed for diagnostic testing.

### 2.2. Preparation of Nano-Niclosamide (Nano-NCL) and Solutions

**Figure 1:**
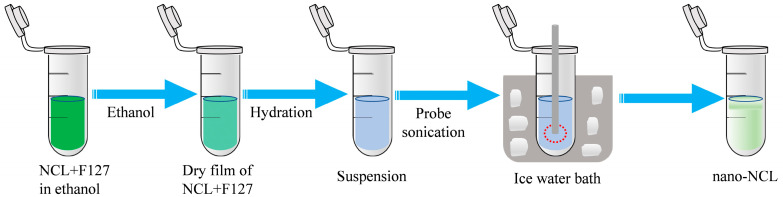
Preparation of nano-NCL. The preparation process primarily involves five steps, as indicated by the arrows: dissolution in ethanol, evaporation, hydration, sonication, and centrifugation. Specifically, NCL and Pluronic F127 were dissolved in ethanol and heated to evaporate the ethanol, and the resulting solid was dispersed in distilled water. After sonication and further treatment in an ice-water bath, the suspension was centrifuged, and the supernatant was collected as the nano-NCL product.

Nano-NCL was prepared based on the method described by Park J et al., which involves crystallization in a surfactant-containing medium [23]. To prepare a 0.3% (3 mg/mL) nano-NCL solution, dissolve 30 mg of NCL and 30 mg of Pluronic F127 in 1 mL of anhydrous ethanol. The solution was then placed in an open container and subjected to anhydrous ethanol evaporation using a water bath at 40 °C under atmospheric pressure for a duration of 30 min. The volume of deionized water used for film hydration was 10 mL. Next, the preparation was carried out in a beaker, and the suspension was subjected to sonication using both bath and probe sonication. The bath sonicator (Heated Ultrasonic Cleaning Machine, SCQ-8201B, Shanghai, China) was operated at a 40% energy level for 10 min with 2 min intervals in an ice-water bath to prevent overheating and break the particles. Finally, centrifuge the suspension at 12,000 rpm for 10 min at a low temperature and collect the supernatant as the nano-NCL product. This procedure yields 10 mL of nano-NCL solution at 0.3% (3 mg/mL) (Figure 1). Dilute the 0.3% nano-NCL solution with distilled deionized water (DDW) in a 1:1 ratio to obtain 0.15% nano-NCL.

The TTO eye pad and Okra eye pad products come with an infused liquid containing active ingredients within their packaging. Under sterile conditions, the infused liquid was carefully extracted from the TTO eye pads and transferred into sterile EP tubes for subsequent acaricidal effect testing. Similarly, the Okra solution was prepared by extracting the liquid from the Okra eye pads under sterile conditions. Both solutions were obtained from the excess liquid in the packaging of the pre-soaked eye pads. A 0.3% F127 dissolved in DDW was used as the vehicle control, while DDW served as the negative control.

### 2.3. Acquisition and Cultivation of Demodex folliculorum

The study followed the tenets of the Declaration of Helsinki and was approved by the Xiamen University Affiliated Xiamen Eye Center Ethics Committee, with approval number XMYKZX-KY-2023-025, dated 21 September 2023. *Demodex folliculorum* specimens were obtained from patients undergoing microscopic examination for mites at the Xiamen University Affiliated Xiamen Eye Center. The procedure involved examining the patients’ eyelid margins with a slit-lamp microscope, identifying eyelashes with collarettes, and rotating them several times using tweezers before extraction. The collected eyelashes were placed on a glass slide for Demodex mites’ assessment. The observation protocol for Demodex mites was adapted from prior research. By adapting a previously established observation protocol, eyelashes with collarettes were systematically scanned under a microscope at 50× magnification to locate all mites [24,25]. Subsequently, the morphology of the identified mites was examined at 100× or 200× magnification to distinguish the mature specimens. The vitality of the mature Demodex mites was assessed by observing leg and chelicerae motility for 1 min, with the mites performing more than 15 movements being classified as having high vigor. Only mature, viable mites were included in the subsequent analysis.

### 2.4. Viability Assessment of Demodex folliculorum

Each group was randomly assigned ten to thirty Demodex mites exhibiting high vitality. A total of 50 µL of the test formulation or solvent was applied to the slide containing the selected mites. The morphology, motility, and survival of the mites were observed at 5-min intervals, with each observation lasting 1 min. The mites were classified as dead when all four pairs of legs and chelicerae ceased movement for two consecutive observation periods. The duration from the application of the drug until the cessation of movement was documented as the survival time.

### 2.5. Nano-NCL Characterization

#### 2.5.1. Particle Size and Zeta Potential

The 0.3% nano-NCL suspension was diluted 100 times with different liquids, including DDW, PBS, and DMEM, and stored at room temperature for up to 20 days. Particle size measurements were performed every 5 days on the diluted solutions. Prior to each measurement, the suspensions were sonicated for 5 min using a probe sonicator to ensure homogeneity. For the particle size analysis, the Zetasizer Nano-ZS90 (Malvern Instruments, Westborough, MA, USA) was used. The temperature during the measurement was controlled at 25 °C, and the suspension was equilibrated for 1 h before the analysis. The zeta potential was directly measured by diluting the 0.3% nano-NCL suspension and F127 100 times with DDW and analyzed at room temperature. The particle size was measured in PBS (1 mM, pH 7.4), and the concentration of the nano-NCL suspension was maintained at 30 µg/mL for all measurements. The zeta potential was directly measured by diluting the 0.3% nano-NCL suspension and F127 to 30 µg/mL with DDW and analyzed at room temperature.

#### 2.5.2. Transmission Electron Microscope (TEM)

The morphology of the nano-NCL was examined using a Hitachi transmission electron microscope (HT-7800, Tokyo, Japan). The 0.3% nano-NCL suspension was diluted 1000 times with DDW before imaging. The samples were mounted on a 400-mesh Cu grid, then stained with 2% phosphotungstic acid for 10 min to enhance contrast. Afterward, the samples were air-dried at room temperature. The imaging was performed under an acceleration voltage of 100 kV, with a magnification of 50,000× for a detailed observation of the nano-NCL particles.

#### 2.5.3. pH Detection

A total of 0.08 g of NaOH was dissolved in 20 mL of DDW to prepare a 0.1 M NaOH solution for the positive control. The 0.3% nano-NCL suspension was diluted to 0.03% with DDW for testing. The pH test paper was immersed in 0.03% nano-NCL or NaOH for 1 s, then removed and left for 30 s to allow the color to stabilize. After stabilization, the color of the test paper was compared with the standard color chart. At 25 °C, the pH meter (Leici, PHS-25, Shanghai, China) was calibrated using pH calibration buffers. The pH of the 0.03% nano-NCL was then detected. Once the pH stabilized, the reading was recorded. The same tube of nano-NCL was placed at room temperature and examined at intervals from 6 h to 24 h.

#### 2.5.4. Bacterial Culture

In a sterile laminar flow cabinet, 50 µL of the 0.3% nano-NCL solution was evenly distributed on the left side, and 50 µL of Escherichia coli was evenly distributed on the right side of a Luria-Bertani (LB) agar plate using a sterile glass spreader. The plate was incubated at 37 °C with 5% CO_2_ for 3 days. After incubation, colony growth was observed and documented by photographing.

#### 2.5.5. High-Performance Liquid Chromatography

The content of NCL was determined using ultra-high-performance liquid chromatography (UHPLC, U3000 RSL). For the sample analysis, 3 mg/mL nano-NCL was diluted to 50 µg/mL with methanol before detection. NCL was dissolved in methanol, and a standard curve was prepared using concentrations ranging from 0.1 µg/mL to 100 µg/mL. The chromatographic conditions were as follows: C18 column (250 × 4.6 mm, particle size: 5 µm); aqueous phase (0.1% formic acid in water); organic phase (methanol); UV detection at 332 nm. The flow rate was 1 mL/min; the column temperature was 35 °C.

The peak areas of the sample and standard solutions were recorded. The standard curve (prepared with NCL concentrations from 0.1 µg/mL to 100 µg/mL) was used to determine the NCL concentration in the sample.

### 2.6. Safety Assessment

C57BL/6 male mice (aged 8 to 10 weeks, weighing 20 to 22 g) were obtained from Shanghai SLAC Laboratory Animal Center (Shanghai, China). This study received approval from the Experimental Animal Ethics Committee of Xiamen University (XMULAC20220258, dated 01 March 2022, Xiamen, Fujian, China) and adhered to the Association for Research in Vision and Ophthalmology (ARVO) guidelines for animal use in ophthalmic and vision research. Solutions of 0.15% and 0.3% nano-NCL were administered as eye drops, with 2 µL delivered to each eye three times daily. After 7 days of treatment, slit-lamp examination and corneal fluorescein staining were conducted. The procedure included capturing slit-lamp images of the murine eyes under white light. Fluorescein sodium strips were immersed in PBS to prepare a fluorescein sodium solution. Subsequently, 1 µL of the solution was applied to the murine eyes, followed by blinking. Finally, slit-lamp images were taken under blue light. Additionally, the corneal epithelial layer, stromal layer, and endothelial layer were scanned using an in vivo confocal microscope (Heidelberg Engineering Inc., Heidelberg, Germany). Carbomer Eye Gel was used as an immersion solution throughout the scan.

### 2.7. Statistical Analysis

A statistical analysis was conducted using GraphPad Prism 8.4.3 (San Diego, CA, USA). Kaplan–Meier curves were generated to assess mite survival distribution, and comparisons between the groups were performed using the log-rank test. Survival times were expressed as a mean ± standard error of the mean (SEM) and analyzed using a one-way ANOVA, with post hoc correction applied via Tukey’s HSD test to adjust for multiple comparisons. A *p*-value < 0.05 was considered statistically significant (* *p* < 0.05, ** *p* < 0.01, *** *p* < 0.001).

## 3. Results

### 3.1. Preparation and Characterization of Nano-Niclosamide

**Figure 2:**
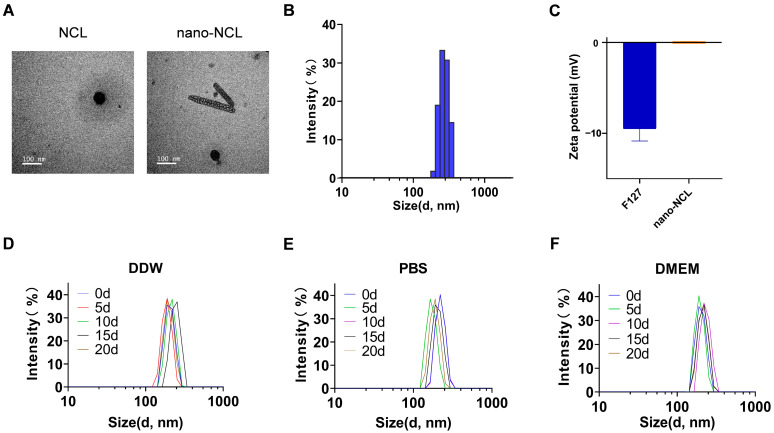
Characterization of nano-NCL. (**A**) Transmission electron microscopy images of niclosamide (left) and nano-NCL (right). Scale bar: 100 nm. (**B**) Particle size distribution of nano-NCL. (**C**) Zeta potential of nano-NCL. (**D**–**F**) Particle size distribution of nano-NCL in DDW, PBS, and DMEM over time (0–20 d).

TEM images (Figure 2A) revealed uniformly rod-shaped particles with smooth surfaces, confirming both the external morphology and structural integrity of the nano-NCL particles. Based on the particle size distribution, the nano-NCL formulation has an average size of approximately 291 nm (Figure 2B), with stable results observed in DDW, PBS, and DMEM over 20 days (Figure 2D–F). As a solvent, the zeta potential of F127 is −10 mV, while the zeta potential of nano-NCL has an average value of 0.27 mV, suggesting that electrostatic stabilization may not be the primary factor responsible for the formulation’s stability (Figure 2C). The concentration of nano-NCL was quantified using high-performance liquid chromatography, and the encapsulation efficiency was calculated to be 40% based on the measured concentration.

**Figure 3:**
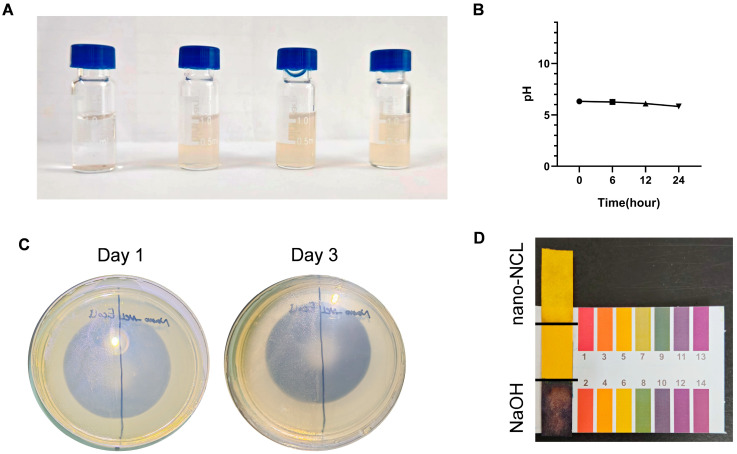
Quality parameter evaluation. (**A**) Images of the nano-NCL solution. From left to right: 0.3% NCL in DDW, 0.15% nano-NCL, 0.3% nano-NCL, and 0.3% nano-NCL stored at room temperature for 3 weeks. (**B**) pH of nano-NCL over time. (**C**) Bacterial culture on LB agar plate: *E. coli* (left) and nano-NCL (right). (**D**) pH test paper detection of nano-NCL.

The pH and sterility of the nano-formulation were also assessed to evaluate its clinical potential. NCL is insoluble in DDW, so 3 mg of NCL would precipitate in DDW. However, the nano-NCL formulation, which enhances the solubility of NCL, remains a stable suspension at a concentration of 0.3% even at room temperature. After 20 days at room temperature, nano-NCL showed no sedimentation or precipitation (Figure 3A). The pH of nano-NCL was stable at around 6, measured with both pH paper and a pH meter (Figure 3B,D). To test sterility, nano-NCL was spread on an LB agar plate, with E. coli as a positive control. After 3 days, no bacterial growth was observed on the nano-NCL side (Figure 3C).

### 3.2. In Vitro Killing Effects of Nano-Niclosamide on Demodex folliculorum

**Figure 4:**
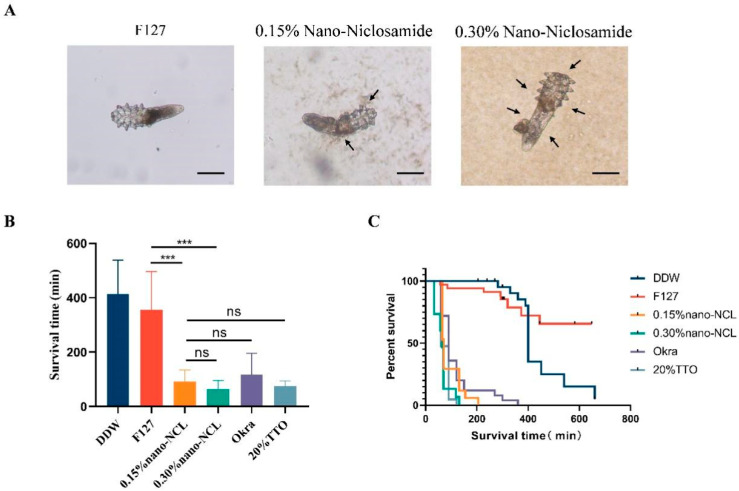
(**A**) The general optical microscope images under 200× magnification of *Demodex folliculorum* in F127, 0.15% nano-NCL, and 0.30% nano-NCL. Scale bar: 100 µm. (**B**) Survival time (min) of *Demodex folliculorum* in the nano-NCL, Okra, and TTO groups. A one-way ANOVA test is used for 0.15% nano-NCL and other groups. *** *p* < 0.001, ns: not significant. (**C**) Kaplan-Meier survival curves of *Demodex folliculorum* under different treatment groups.

When treated with F127 (vehicle control), 0.15% nano-NCL, or 0.30% nano-NCL, optical microscope images revealed that the nanoparticles were either in close proximity to or surrounding the mites. At a concentration of 0.15% nano-NCL, the nanoparticles adhered to the mite’s surface, while the coverage significantly increased at 0.30% (Figure 4A). In the 0.15% nano-NCL group, 70.6% of the mites died within 2 h, with an average survival time of 92 ± 41 min. In the 0.30% nano-NCL group, 92.3% of the mites died, with an average survival time of 65 ± 30 min. However, the difference between these two groups was not statistically significant (*p* > 0.05), indicating no clear concentration-dependent effect. The TTO group had a slightly higher mortality rate (95.3%) and longer survival time (76 ± 18 min) than the 0.30% nano-NCL group. The Okra group had a lower mortality rate (64%) and a longer survival time (118 ± 77 min) (Table 1). No statistically significant difference in the survival time was observed between the groups (Figure 4B). The survival curves of Demodex in Figure 4C demonstrate that both 0.15% and 0.30% nano-NCL drastically decreased the survival probability of Demodex over time, outperforming the vehicle control and DDW. According to the log-rank test (Table 2), there was no significant difference between the two nano-NCL concentrations and the TTO and Okra groups (Table 1). Consequently, the effectiveness of TTO and Okra was comparable to that of nano-NCL, but it did not outperform nano-NCL.

### 3.3. Safety Evaluation of Nano-NCL

**Figure 5:**
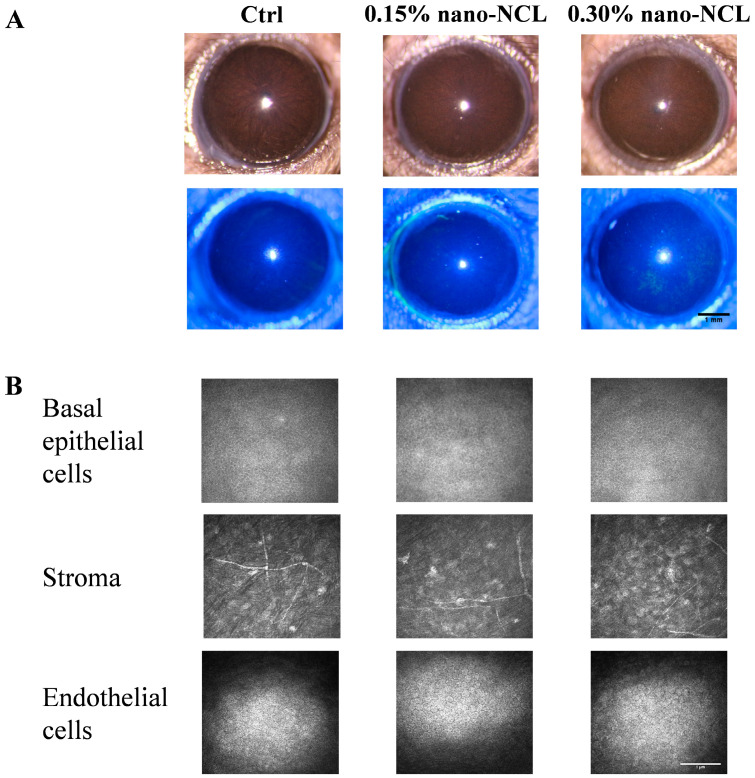
Safety assessment of nano-NCL. (**A**) Representative images of fluorescein sodium staining in Ctrl, 0.15% nano-NCL, and 0.3% nano-NCL. Scale bar: 1 mm (**B**) In vivo confocal microscopy images from different layers of the cornea after 0.15% nano-NCL and 0.3% nano-NCL eye drops administration twice a day for 7 days. Scale bar: 1 µm.

After topical administration of two concentrations of nano-NCL in mice for seven days, an initial safety assessment of the ocular surface revealed that the 0.3% nano-NCL group exhibited slight fluorescein staining, while the 0.15% group showed no significant staining (Figure 5A). In vivo confocal microscopy imaging of different corneal layers indicated that the 0.3% nano-NCL group showed slight infiltration of inflammatory cells in the superficial stromal layer, while the 0.15% treatment group exhibited no significant inflammatory infiltration (Figure 5B). No notable structural damage was observed in either the corneal epithelium or endothelium.

## 4. Discussion

Demodex blepharitis is a chronic lid margin disease with an increasing prevalence on the ocular surface [26,27]. Additionally, Demodex blepharitis is often intertwined with dry eye disease [28]. Considering the direct and indirect role of Demodex in the development of ocular surface diseases, such as blepharitis and dry eye, developing a safe and targeted eye drop therapy is scientifically justified and necessary. NCL was originally applied in humans for its efficacy in eliminating tapeworms and has a well-established history as an antiparasitic agent [29]. In our previous study, we found that 0.5% NCL effectively eliminated mites within 40 min in vitro, demonstrating strong acaricidal activity. To enhance the solubility of NCL, we previously incorporated TWEEN 80 and PEG 300 as solubilizers [30]. However, a high proportion of TWEEN 80 not only exhibited inherent mite-inhibitory effects but also caused ocular surface irritation [30,31,32]. To improve the ocular safety of NCL eye drops, we developed nano-NCL. Although mites exhibited a longer average survival time in the nano-NCL formulation compared to the conventional NCL solution, nano-NCL demonstrated superior ocular tolerability, providing a promising approach for mite eradication via ophthalmic administration.

To enhance the solubility and bioavailability of NCL for ophthalmic use, nano-NCL was developed and evaluated for its in vitro mite-killing efficacy. Firstly, the nano-NCL has a length of approximately 291 nm and remains stable at room temperature for more than two weeks, which contributes to the prolonged stability and effectiveness of the drug. Additionally, the formulation maintains a stable pH of 6 and shows no signs of bacterial growth, confirming its sterility and appropriateness for clinical application. Both concentrations of nano-NCL (0.15% and 0.30%) significantly reduced Demodex survival time and survival properties in vitro compared with control groups. Additionally, 0.15% nano-NCL has shown promising ocular surface safety in preliminary evaluations. There were no differences between the NCL groups and the TTO and Okra groups. This study is the first to apply a nano-crystal formulation of NCL for ocular surface diseases, and its in vitro anti-Demodex activity was preliminarily evaluated. Nano-NCL demonstrated mite-killing efficacy comparable to clinically used treatments, such as Okra and 20% TTO, while demonstrating good safety on the ocular surface. Overall, nano-NCL emerges as a promising candidate for the treatment of Demodex blepharitis.

Although the exact mechanisms responsible for nano-NCL’s anti-Demodex activity have not been fully uncovered, it is plausible that its nano-formulation enhances the drug’s interaction with Demodex mites, facilitating greater penetration and improved bioavailability at the site of infestation. The nanoscale particles may allow better adhesion to the mites and deeper penetration into the follicular or glandular structures where Demodex resides, leading to quicker immobilization and death [33]. Additionally, the formulation may disrupt the biological functions of the mites, such as respiration and nutrition, thereby accelerating mortality. This hypothesis is consistent with the survival data, as nano-NCL demonstrated swift reductions in Demodex survival time and survival properties, indicating that the formulation’s distinctive physicochemical properties play a pivotal role in its efficacy. Interestingly, no significant difference was observed in efficacy between the two concentrations of nano-NCL (0.15% and 0.30%). This suggests that 0.15% nano-NCL is enough to achieve optimal anti-Demodex effects, reducing unnecessary exposure to higher doses while maintaining effectiveness.

A 20% concentration of TTO is currently a widely used treatment for Demodex-related blepharitis [34]. Although the precise mechanism of its antiparasitic activity remains unclear, its miticidal effect is partially attributed to the anticholinesterase action of T4O, 1,8-cineole, γ-terpinene, α-terpinene, and ρ-cymene, which induces fatal muscle contraction and spastic paralysis in the parasite [25,35]. Nano-NCL’s comparable efficacy, coupled with its reduced risk of irritation, positions it as a promising candidate for Demodex treatment, especially for patients who are sensitive to TTO. Additionally, Okra extract has emerged as a novel therapeutic option for Demodex-related blepharitis. A reported clinical trial indicated that Okra patches effectively eradicated ocular Demodex and alleviated Demodex-related blepharitis symptoms with reduced irritation compared to TTO [13]. In the current study, Okra’s impact on Demodex was somewhat less significant compared to nano-NCL, particularly in terms of reducing survival time and cumulative mortality rates. In this study, both TTO and Okra were derived from the storage solution of eye pads, rather than being specifically formulated for eye drop application. As such, the nano-NCL formulation provides a safer and more convenient method for treating Demodex blepharitis via eye drops. Additionally, the mortality observed in the control group can be attributed to the limited survival of Demodex mites on glass slides. These mites rely on eyelash follicles for nourishment and an appropriate environment. Without access to this natural habitat, they are unable to survive for prolonged periods in vitro. Consequently, the mites exhibited natural mortality, which highlights the challenges of maintaining Demodex mites in artificial conditions outside their typical host environment.

An evaluation of ocular surface safety in mice following the instillation of nano-NCL revealed that the 0.15% nano-NCL formulation exhibited superior safety. Furthermore, the insignificant differences between the 0.15% and 0.30% nano-NCL concentrations underscore the feasibility of utilizing lower-dose formulations in clinical settings, which could decrease both costs and the likelihood of adverse reactions. Consequently, 0.15% nano-NCL stands out as a robust candidate for future clinical trials and potential commercialization.

## 5. Conclusions and Limitations

In conclusion, nano-NCL, particularly at 0.15%, demonstrates potent anti-Demodex activity, comparable to that of 20% TTO and Okra. Its favorable safety profile, combined with its rapid and effective eradication of Demodex, positions it as a promising therapeutic option for the management of Demodex-related ocular disease. While the current study offers compelling evidence for nano-NCL’s effectiveness against Demodex, additional research is necessary to comprehensively understand its mechanism of action. In addition, in vivo studies assessing the safety and long-term effects of nano-NCL on the ocular surface are essential for further clinical utility. Comparative studies involving larger sample sizes and a diverse range of patient populations are essential for validating the present findings and establishing nano-NCL as a standard-of-care treatment for Demodex infestations.

## Figures and Tables

**Table 1:** Results of in vitro test of Demodex in contact with different treatments.

Group	Number	Min (min)	Max (min)	Mean (SD) (min)	Mite Mortality in 2 h (%)
DDW	23	282	660	414 (121)	0
F127	34	227	445	360 (138)	5.7
0.15% nano-NCL	17	65	205	92 (41)	70.6
0.30% nano-NCL	13	33	131	65 (30)	92.3
20% TTO	21	60	120	76 (18)	95.3
Okra	25	60	360	118 (77)	64

**Table 2:** The comparison of the survival distribution between the pairs using the log-rank test.

Group A	Group B	*p*-Value
0.15% nano-NCL	DDW	<0.0001
0.15% nano-NCL	F127	<0.0001
0.3% nano-NCL	DDW	<0.0001
0.3% nano-NCL	F127	<0.0001
0.3% nano-NCL	0.15% nano-NCL	0.0600
20% TTO	DDW	<0.0001
20% TTO	F127	<0.0001
20% TTO	0.15% nano-NCL	0.0700
20% TTO	0.3% nano-NCL	0.6300
20% TTO	Okra	0.0047
Okra	DDW	<0.0001
Okra	F127	<0.0001
Okra	0.15% nano-NCL	0.3500
Okra	0.3% nano-NCL	0.0040
F127	DDW	0.0200

## Data Availability

Data will be made available on request.
